# Hypoglycemic coma due to insulin autoimmune syndrome induced by methimazole: A rare case report

**DOI:** 10.3892/etm.2014.1964

**Published:** 2014-09-15

**Authors:** YIYI ZHANG, TIEYUN ZHAO

**Affiliations:** Department of Endocrinology and Metabolism, West China Hospital, Sichuan University, Chengdu, Sichuan 610041, P.R. China

**Keywords:** hypoglycemia, insulin autoimmune syndrome, insulin autoantibody, methimazole

## Abstract

Insulin autoimmune syndrome (IAS) is a rare cause of hypoglycemia characterized by the presence of insulin-binding autoantibodies and fasting or late postprandial hypoglycemia. The number of reports on the association of human leukocyte antigen (HLA) genotype with this disease in adolescents in China is limited. This is the case report of a 17-year-old female patient with Graves’ disease who was treated with methimazole (MTZ). After 4 weeks of continuous MTZ treatment, the patient suffered an episode of unconsciousness during the late postprandial phase and was admitted to the hospital, where the blood glucose level was found to be 2.88 mmol/l. The symptoms were relieved following intravenous glucose administration. Imaging studies of the pancreas were unremarkable, but the laboratory investigations on admission revealed high serum levels of total insulin, associated with relatively low levels of free insulin and markedly elevated insulin autoantibody (IAB) levels. HLA testing revealed DRB1^*^0406/0901 and the patient discontinued MTZ and was prescribed propylthiouracil. During the long-term follow-up, the total insulin and IAB levels gradually declined. There was no other episode of hypoglycemia. Therefore, in adolescents with Graves’ disease receiving antithyroid treatment with MTZ who experience hypoglycemia, the IAB levels should be assessed to exclude or confirm IAS as the underlying cause.

## Introduction

Insulin autoimmune syndrome (IAS), or Hirata’s disease, is a rare condition characterized by the combination of recurrent, severe spontaneous hypoglycemia, high concentration of total immunoreactive insulin (IRI) and the presence of autoantibodies to insulin in patients who have not received insulin injections. Since Hirata *et al* first described the disease (1), there have been a total of 330 reported cases of IAS over the last 37 years in Japan (2), where IAS is the third leading cause of spontaneous hypoglycemia, following insulinoma and diffuse islet-cell hyperplasia (nesidioblastosis). IAS is more common among patients aged >40 years, with reports of this sundrome in the pediatric age group being extremely rare. The peak age of onset was reported to be 60–69 years for both genders (2) and the incidence of IAS is lower in countries outside Japan. The number of reports of this disorder in China and, particularly, in adolescents, is limited. In addition, reports on IAS-related human leukocyte antigen (HLA) genotype are rare, with only 84 cases reported in China by late 2012 (3). This is the rare case report of a methimazole (MTZ)-associated IAS in a Chinese female adolescent patient.

## Case report

A 17-year-old female patient from Southwest China was referred to the local hospital due to recurrent episodes of weariness, sweating, palpitations and weight loss of ~5 kg within a few months. The patient’s thyroid-stimulating hormone was 0.011 mU/l (reference range, 0.27–4.2 mU/l) and her free thyroxine was 100 pmol/l (reference range, 12.0–22.0 pmol/l). The patient was diagnosed with hyperthyroidism and antithyroid treatment with MTZ (10 mg, three times daily) was initiated. After 2 weeks of continuous MTZ administration, the patient experienced dizziness, weakness, cold sweats, palpitations and tremor ~2 h after breakfast. The symptoms disappeared following ingestion of food (bread, milk). Four weeks later, the patient experienced palpitations and sweating followed by unconsciousness during the late postprandial phase (3 h after the ingestion of food). The patient was immediately admitted to a local hospital and hypoglycemia was diagnosed on the basis of a blood glucose concentration of 2.88 mmol/l and symptom relief following intravenous glucose administration; however, the cause of hypoglycemia had not been identified. The patient was referred to our hospital for further assessment and treatment. The physical examination on admission revealed a chronically ill appearance, with a weight of 65 kg, a height of 170 cm (BMI, 22.5 kg/m^2^) and blood pressure of 110/75 mmHg. There was no exophthalmos and no thyroid enlargement, although the thyroid was diffusely tender on palpation. The other systematic examinations were normal. The patient had no history of diabetes mellitus, infectious diseases, insulin usage or intake of oral hypoglycemic agents. The laboratory tests revealed that the thyroid-stimulating hormone level was 0.010 mU/l (reference range, 0.27–4.2 mU/l), the free thyroxine was 91.54 pmol/l (reference range, 12.0–22.0 pmol/l) and the thyrotrophin receptor antibody was 27.48 IU/l (reference range, <3 IU/l), reflecting uncontrolled Graves’ disease. The patient’s fasting plasma glucose was 4.62 mmol/l, the glycosylated hemoglobin was 5.6%, the fasting serum total IRI was >1,000 mU/ml (reference range, 1.5–15 mU/l) and the free serum C-peptide was 2.04 nmol/ml (reference range, 0.48–0.78 nmol/ml). The haemoglobin concentration, leukocyte count, platelet count, erythrocyte sedimentation rate and liver, renal and adrenal function tests were all normal. There was no space-occupying lesion in the pancreas on abdominal computed tomography.

**Figure 1:**
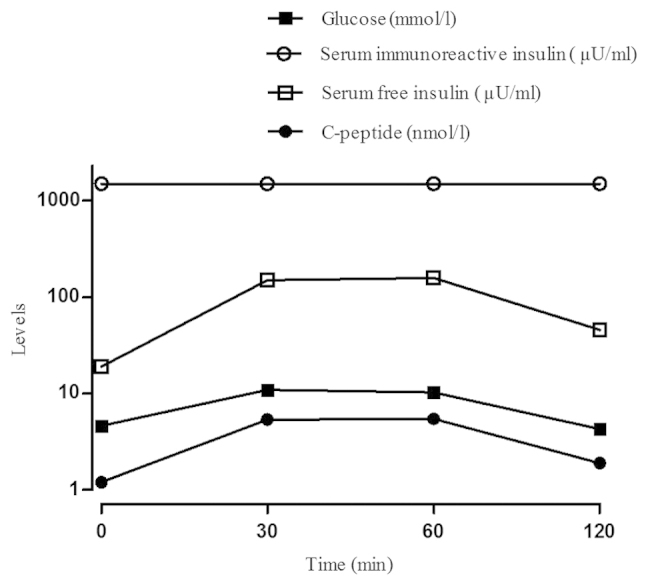
Results of the oral glucose tolerance test (glucose level at 0, 0.5, 1, and 2 h after a 75 g glucose drink) and the insulin release test (serum immunoreactive insulin level) and synchronized free insulin and C-peptide levels on admission.

The laboratory tests revealed disproportionately increased serum total IRI and C-peptide levels and anti-islet β-cell autoantibodies were evaluated. The islet cell antibody (ICA) and glutamic acid decarboxylase antibody (GADA) tests were negative, but the insulin autoantibody (IAB) level was 2.40 U/ml (reference range, negative). To further determine the association between the serum concentrations of total IRI, free insulin, glucose and C-peptide, the patient underwent an oral glucose tolerance test, an insulin release test, the synchronized free insulin and C-peptide levels were evaluated and the polyethelene glycol (PEG) precipitation method was employed to remove IABs prior to free insulin measurement to exclude interference. The results revealed that total IRI levels were significantly elevated at all time points, unlike C-peptide, which remained elevated while plasma glucose began to decline during the oral glucose tolerance test, suggesting a delayed clearance of insulin. Free insulin levels were marginally increased at 30 min, but were significantly lower compared to the total IRI, followed by a slow decline, beginning to decline significantly by 90 min, with plasma glucose levels fluctuating between 4.52 and 10.9 mmol/l after a 75 g oral glucose load (Fig. 1). Serum total IRI and free insulin levels were significantly increased disproportionately, which indicated that hypoglycemia may be secondary to MTZ-induced IAS. The patient was advised to discontinue MTZ and was prescribed propylthiouracil (PTU) 100 mg three times daily.

**Figure 2:**
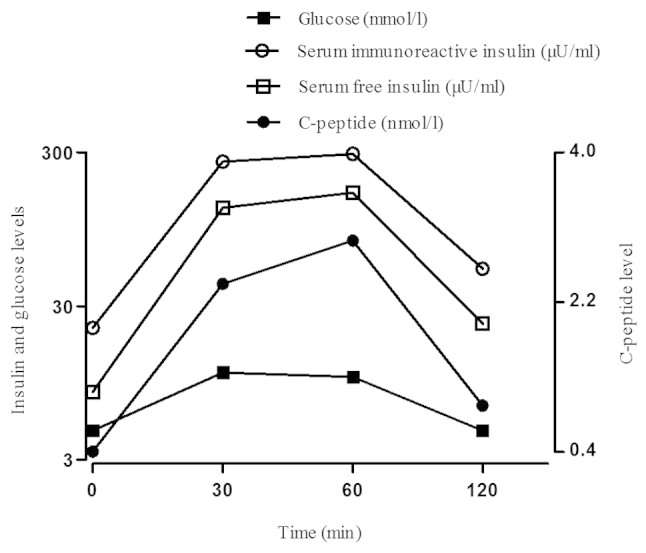
Results of the oral glucose tolerance test (glucose level at 0, 0.5, 1, and 2 h after a 75 g glucose drink) and the insulin release test (serum immunoreactive insulin level) and synchronized free insulin and C-peptide levels 5 months after PTU was prescribed and the result of the blood test for IAB came back negative.

The patient continued the treatment protocol after discharge and did not experience any further hypoglycemic episodes over the following 5 months. The symptoms caused by hyperthyroidism had also improved at the follow-up visit after 5 months, the thyroid stimulating hormone level was 0.027 mU/l and the free thyroxine level was 17.72 pmol/l. The oral glucose tolerance and insulin release tests revealed that the total IRI levels had decreased markedly compared to those on admission, but remained elevated above baseline. The synchronized free insulin and C-peptide levels had returned to normal (Fig. 2). During long-term follow-up, the IAB levels gradually declined and were not detectable after 5 months of PTU administration (Table I). The patient’s HLA typing by polymerase chain reaction-sequence based typing revealed the DRB1^*^0406/0901 genotype.

## Discussion

Our patient had received MTZ for hyperthyroidism for ~2 weeks prior to the first hypoglycemic attack. There was no exposure history to insulin or insulin-stimulating drug therapy; therefore, oral hypoglycemic agents or exogenous insulin-induced causes of hypoglycemia were excluded. The insulin release test revealed that serum total IRI and free insulin levels were significantly increased disproportionately, reflecting the separation of the two insulins. The serum IRI and C-peptide levels were not synchronized and serum IAB was increased. In addition, there were no pancreatic abnormalities on abdominal computed tomography and the ICA and GADA tests were negative. No underlying autoimmune disorders or other causes of IAS could be identified, excluding the possibility that the hypoglycemia was caused by exogenous insulin increase. Therefore, it was concluded that the hypoglycemia was due to IAB production induced by MTZ treatment.

The cause of IAS is heterogeneous and has not been fully elucidated. A previous study including 380 Japanese IAS patients reported that ~50% of the patients had received drugs with sulfhydryl groups (2), such as MTZ (most common) (4), thiopronin, penicillamine, glutathione, as well as other drugs, including hydralazine, procainamide and isoniazid. It has been hypothesized that sulfhydryl group drugs may cleave the disulfide bond of the insulin molecule *in vivo* and enhance its immunogenicity, which may lead to the production of IABs (5). In our patient, the history of exposure to MTZ supported an etiological role for drugs with sulfhydryl group in the development of IAS.

Previous studies have divided IAB into two types, namely low-affinity/high-capacity and high-affinity/low-capacity types (6,7). Eguchi *et al* (8) reported that the IAB produced in patients with IAS is of the low-affinity/high-capacity type, as demonstrated on the Scatchard plot. In our patient, the symptoms of hypoglycemia occurred 2–3 h postprandially, as a large number of insulin molecules released after food ingestion may bind to these low-affinity/high-capacity IABs, which prevent insulin from acting. Therefore, elevated blood glucose levels further stimulate islet β cells to release more insulin, which also binds to the IABs. The bound insulin may readily dissociate from the IABs, resulting in a rapid increase in free insulin levels and consequent hypoglycemia.

A characteristic of this patient’s presentation was the strikingly high levels of total IRI (9). Insulin was measured by radioimmunoassay; IAB and anti-IAB reagents competitively bound, which caused spurious hyperinsulinemia. The PEG precipitation method was employed to remove IABs prior to free insulin measurement in order to exclude interference. The free insulin levels were marginally increased, but were significantly lower compared to total IRI, reflecting the separation of free insulin and total IRI. Human proinsulin C-peptide is a cleavage product of insulin secretion in the β cells of the islets of Langerhans and is released in amounts equal to insulin into the portal circulation (10). C-peptide levels may be associated with the secretion of islet β cells (11). Since specific IABs may crossreact with proinsulin and C-peptide levels were moderately elevated, the serum C-peptide levels actually did not accurately reflect the secretion of islet β cells. Despite the concordant elevation of insulin and C-peptide levels in our tests, as the proportion of proinsulin in the blood was low, a discrepancy between excessively high IRI and only moderately elevated C-peptide levels was obvious.

The majority of IAS cases described in Asians exhibit a strong correlation with certain HLA systems, suggesting the existence of a predisposing genetic component. It is noteworthy that HLA-DRB1^*^0406 is quite prevalent among East Asian patients, but Caucasian patients mainly express HLA-DRB1^*^0403 (12). In fact, Japanese IAS patients were found to be DR4-positive in 96% (49/51) of cases and 82% (42/51) of cases possessed DRB1^*^0406 in the polyclonal type (13). A previous study confirmed that the product of DRB1^*^0406 is an insulin antigen-presenting major histocompatibility complex molecule (14); therefore, populations with a higher prevalence of DRB1^*^0406 are at higher risk of developing IAS. The number of reports of the association of this disorder with HLA genotype in China is limited, with a lack of large-scale research data. By late 2012, only one case of a Chinese patient with DRB1^*^0406 had been reported (15). The adolescent patient described in this case report was HLA-DRB1^*^0406/0901, which supports an common genetic and immunological basis of IAS.

In Japan, IAS is reportedly the third leading cause of spontaneous hypoglycemia, in addition to insulinoma and diffuse islet-cell hyperplasia (nesidioblastosis). It is generally considered that insulinoma may also be characterized by low blood glucose and high insulin levels (16). As in several cases of insulinoma without a reliable tumor localization prior to surgery, small tumors are often only identified by an experienced surgeon (17), it was necessary to evaluate IAB to avoid unnecessary surgery (5,18). In addition, these findings almost certainly excluded the possibility of occult insulinoma or nesidioblastosis. It was previously suggested that insulin levels >1,000 pmol/l argue against insulinoma and raise the suspicion of IAS (17). There were no pancreatic abnormalities on abdominal computed tomography, the IAB test was positive, an insulin release test exhibited the separating phenomenon of free insulin and total IRI and the process of the disease described in this case report was self-limiting. The patient was advised to discontinue MTZ and supportive treatment was provided. In addition, there was no other episode of hypoglycemia. Therefore, insulinoma was excluded as the cause of hypoglycemia.

Approximately 80% of the Japanese IAS patients reported between 1970 and 2007 experienced a spontaneous remission without special treatment (2). Spontaneous remission may develop in <3 months. Following emergency treatment of hypoglycemia, an α-glucosidase inhibitor may be prescribed in combination with dietary control to decrease endogenous insulin secretion (19). It has been reported that, in patients with very high IAB concentrations, large dose of chloroquine, cyclophosphamide and corticosteroids may significantly lower plasma IAB concentrations. It has also been reported that plasma exchange successfully decreased the IAB concentration in a patient with severe, repeated episodes of hypoglycemia (20,21). For refractory patients with frequent episodes of hypoglycemia, partial pancreatectomy may be feasible, but is now rarely used in clinical practice (6). In the present case, antithyroid treatment with MTZ was discontinued after admission and PTU was prescribed, with no further episodes of hypoglycemia.

Although hypoglycemia caused by MTZ-induced IAS is rare in adolescents with Graves’ disease, clinicians should be aware of this possible etiology. For Graves’ disease patients receiving antithyroid treatment with MTZ who experience hypoglycemia and whose hypoglycemia cannot be explained by other causes, IAS must be considered in order to avoid undue pancreatic surgery and IAB levels should be assessed to exclude or confirm this as the underlying cause. If MTZ-induced IAS is confirmed, the patient should be advised to avoid the use of MTZ thereafter.

## Figures and Tables

**Table I:** Anti-islet β-cell autoantibody tests on follow-up visits.

Autoantibodies (U/ml)	2 weeks	2 months	5 months	8 months
ICA (reference range, negative)	Negative	Negative	Negative	Negative
GADA (reference range, <1.05)	Negative	1.02	1.01	0.94
IAB (reference range, negative)	2.04	2.13	Negative	Negative

ICA, islet cell antibody; GADA, glutamic acid decarboxylase antibody; IAB, insulin autoantibody.

## References

[1] Hirata Y, Ishizu H, Ouchi N (1970). Insulin autoimmunity in a case of spontaneous hypoglycemia. J Jpn Diab Soc.

[2] Uchigata Y, Hirata Y, Eisenbarth GS (2011). Insulin autoimmune syndrome (Hirata disease). Immunoendocrinology: Scientific and Clinical Aspects.

[3] Kai L, Xinguo H, Jun S (2011). Clinical characteristics of insulin autoimmune syndrome in Chinese patients. Chin J Intern Med.

[4] Lupsa BC, Chong AY, Cochran EK (2009). Autoimmune forms of hypoglycemia. Medicine (Baltimore).

[5] Uchigata Y, Hirata Y, Eisenbarth GS (1999). Insulin autoimmune syndrome (IAS, Hirata disease). Molecular Mechanisms of Endocrine and Organ Specific Autoimmunity.

[6] Kim MR, Sheeler LR, Mansharamani N (1997). Insulin antibodies and hypoglycemia in diabetic patients. Can a quantitative analysis of antibody binding predict the risk of hypoglycemia?. Endocrine.

[7] Brooks-Worrell BM, Nielson D, Palmer JP (1999). Insulin autoantibodies and insulin antibodies have similar binding characteristics. Proc Assoc Am Physicians.

[8] Eguchi Y, Uchigata Y, Yao K (1994). Longitudinal changes of serum insulin concentration and insulin antibody features in persistent insulin autoimmune syndrome (Hirata’s disease). Autoimmunity.

[9] Gomez Cruz MJ, Jabbar M, Saini N (2012). Severe hypoglycemia secondary to methimazole-induced insulin autoimmune syndrome in a 16 year old African-American male. Pediatr Diabetes.

[10] Rubenstein AH, Clark JL, Melani F (1969). Secretion of proinsulin C-peptide by pancreatic beta cells and its circulation in blood. Nature.

[11] Lohmann T, Kratzsch J, Kellner K (2001). Severe hypoglycemia due to insulin autoimmune syndrome with insulin autoantibodies crossreactive to proinsulin. Exp Clin Endocrinol Diabetes.

[12] Uchigata Y, Hirata Y, Omori Y, Iwamoto Y, Tokunaga K (2000). Worldwide differences in the incidence of insulin autoimmune syndrome (Hirata disease) with respect to the evolution of HLA-DR4 alleles. Hum Immunol.

[13] Cavaco B, Uchigata Y, Porto T (2001). Hypoglycaemia due to insulin autoimmune syndrome: report of two cases with characterisation of HLA alleles and insulin autoantibodies. Eur J Endocrinol.

[14] Murakami M, Mizuide M, Kashima K (2000). Identification of monoclonal insulin autoantibodies in insulin autoimmune syndrome associated with HLA-DRB1*0401. Horm Res.

[15] Jianlin D, Changc L, lin G (2001). Case report: insulin autoimmune syndrome induced by methimazole. Chin J Intern Med.

[16] Moreira RO, Lima GA, Peixoto PC, Farias ML, Vaisman M (2004). Insulin autoimmune syndrome: case report. Sao Paulo Med J.

[17] Virally ML, Guillausseau PJ (1999). Hypoglycemia in adults. Diab Metab.

[18] Basu A, Service FJ, Yu L (2005). Insulin autoimmunity and hypoglycemia in seven white patients. Endocr Pract.

[19] Schlemper RJ, Uchigata Y, Frölich M, Vingerhoeds AC, Meinders AE (1996). Recurrent hypoglycaemia caused by the insulin autoimmune syndrome: the first Dutch case. Neth J Med.

[20] Yaturu S, DePrisco C, Lurie A (2004). Severe autoimmune hypoglycemia with insulin antibodies necessitating plasmapheresis. Endocr Pract.

[21] Greenfield JR, Tuthill A, Soos MA (2009). Severe insulin resistance due to anti-insulin antibodies: response to plasma exchange and immunosuppressive therapy. Diabet Med.

